# Supplementary material for: SARS-CoV-2 seroprevalence among patients with severe mental illness: A cross-sectional study
Source: PLoS One. 2022 Mar 1;17(3):e0264325. doi: 10.1371/journal.pone.0264325 (PMC8887729; doi:10.1371/journal.pone.0264325)
Supplement: Attachment — Submitted filename: Rebuttal letter 02.02.22.docx [file pone.0264325.s002.docx]

**REBUTTAL LETTER_PONE-D-21-26611R1**

We thank all Reviewers for once again carefully revising our manuscript and for raising some important and relevant questions. Please find our specific comments below. Thank you.

6. Review Comments to the Author

Please use the space provided to explain your answers to the questions above. You may also include additional comments for the author, including concerns about dual publication, research ethics, or publication ethics. (Please upload your review as an attachment if it exceeds 20,000 characters)

Reviewer #1: The word adjusted could still be used as alternative to the word corrected.

Author response: revised accordingly. Thank you for the comment.

For Table 1 & 2, chi-squares test where applicable could still be utilized provided the assumptions of the statistical test are fulfilled/met. Please check the assumptions and also recheck the p values. If Fisher's Exact test is used, one sided or two sided p value to be stated.

Author response: Good point. We have now added the sentence: “P-values for Fisher’s exact tests were two-sided”.

Reviewer #2: Revisions were adequate Revisions were adequate Revisions were adequate Revisions were adequate Revisions were adequate

Reviewer #3: (No Response)

Reviewer #4: I thank the authors for their responses to comments. Nevertheless, I have some other comments.

Introduction
If you want to use the acronym SMI, it is best to associate it with severe mental illness as in the abstract. Otherwise use SMD for severe mental disorders.

Author response: Thank you for your observation. You are completely right. Severe mental disorders have now been changed to severe mental illness.

Methods - Statistical analysis
Due to the small number of expected subjects (<5) in some categories, and the highly unbalanced marginal
sums of the actual data set, Pearson’s chi-square test cannot be applied. It is better to use Fisher’s exact test.
Author response: Fisher’s exact test could not be made with too many groups or observations.

Reviewer comment : The extension of Fisher's exact test to the case where the two variables have any finite number of modalities, but greater than two, was first performed by G. H. Freeman and J.H. Halton in 1951. This test is sometimes referred as the Freeman-Halton or Fisher-Freeman-Halton test. So it is possible to calculate Fisher’s exact test with more than 2 modalities in one variable.
It is written in Table 1 that Ficher’s exact test was used. I think this is not the case for Diagnosis according the author response.

Author response: We thank you for the comment and apologize for the misunderstanding regarding the statistical analyses used in the manuscript. After the first comment wisely suggesting Fisher’s exact test as test when comparing categorical demographics (in table 1 and table 2), the authors applied this test together with an unpaired t-test (for continuous demographics). The authors recognize that this was not clearly stated in our responses to the review comments. As written in table 1 and table 2, and the statistical analyses section, Fisher’s exact tests were used.

Results
It is possible to have standardized decimal point in p value.
0.35 Could be witten 0.350.

Author response: All p-values are now standardized to contain three decimals.

There is a confusion in the article between total population (for me the 7310 subjects) and the study population (for me the 1258 subjects
Table 1: change the Table title by « Baseline characteristics of study population according to SARS-Cov-2 serology
Table 2: change the Table title by « Baseline characteristics of total population by study participation

Author response: Thank you. This has now been corrected as suggested.
